# TNF-α Activates NF-κB Signalling Pathway in MG-63 Cells on Titanium and Zirconia Implant Surfaces

**DOI:** 10.3390/ma18040884

**Published:** 2025-02-18

**Authors:** Khaled Mukaddam, Sabrina Ruggiero, Steffen M. Berger, Dietmar Cholewa, Gabriela Dekany, Andreas Bartenstein, Milan Milošević, Sebastian Kühl, Michael M. Bornstein, Farah Alhawasli, Elizaveta Fasler-Kan

**Affiliations:** 1 Department of Oral Surgery, University Center for Dental Medicine Basel (UZB), University of Basel, Mattenstrasse 40, CH-4058 Basel, Switzerland; khaled.mukaddam@unibas.ch (K.M.);; 2 Department of Pediatric Surgery, Children’s Hospital, Inselspital Bern, University of Bern, Freiburgstrasse 15, CH-3010 Bern, Switzerland; 3 Department of Oral Health & Medicine, University Center for Dental Medicine Basel (UZB), University of Basel, Mattenstrasse 40, CH-4058 Basel, Switzerland; 4 Department of Biomedicine, University of Basel, Hebelstrasse 20, CH-4031 Basel, Switzerland

**Keywords:** nuclear factor kappa B (NF-κB), tumour necrosis factor alpha (TNF-α), signalling pathways, MG-63 cells, zirconia, titanium

## Abstract

Dental implant therapy is a widely used clinical procedure for restoring missing teeth in patients. Zirconia implants were introduced as an alternative to titanium implants due to their excellent biocompatibility and esthetic properties. The nuclear factor kappa B (NF-κB) signalling pathway is responsible for multiple aspects of innate and adaptive immune functions and serves as a significant and crucial mediator of inflammatory processes. The dysregulation of NF-κB activation induces pathological processes in multiple diseases. The purpose of this study was to investigate the activation of the NF-κB pathway upon stimulation with tumour necrosis factor (TNF)-α in osteoblast-like cells (MG-63) cultured on zirconia surfaces in comparison to titanium surfaces. Several methods such as immunoblot, immunofluorescence, MTT assay, and flow cytometry were used in this study. We observed that human recombinant TNF-α caused a strong activation of the NF-κB pathway on both zirconia and titanium discs and in wells without any discs. This activation was marked by the upregulation of MHC class I proteins in MG-63 cells grown on both titanium and zirconia discs; however, there was no effect on MHC class II protein expression. In summary, the present study has shown that TNF-α stimulation equally activates the NF-κB pathway in MG-63 cells cultured on both titanium and zirconia surfaces.

## 1. Introduction

In dentistry, the choice of implant material and its surface properties were identified as a vital factor in achieving optimal osseointegration and long-term success [1,2]. For several decades, titanium has been regarded as the gold standard for dental implants or healing abutments [3,4]; however, recently, zirconia became a noticeable alternative with potential benefits in reducing peri-implant infections in patients [5,6,7]. With a success rate of over 97% after five years, zirconia implants have become an important alternative to titanium implants [8,9]. The comparison between zirconia and titanium and their respective influence on the immune response however requires further investigation.

The nuclear factor kappa B (NF-κB) family in mammals is composed of five related transcription factors, namely p50, p52, RelA (p65), c-Rel, and RelB [10,11]. These transcription factors share high homology through a 300 amino acid N-terminal DNA binding/dimerization domain, called the Rel homology domain (RHD). The RHD serves as a platform for the formation of both homo- and heterodimers among NF-κB family members, allowing them to bind to promoter and enhancer regions of genes and regulate their expression [11].

RelA, c-Rel, and RelB contain C-terminal transcriptional activation domains (TADs), which enable them to activate target gene expression, but in contrast, p50 and p52 lack C-terminal TADs. Hence, p50 and p52 homodimers repress transcription unless they are bound to a protein containing a TAD, such as RelA, c-Rel, or RelB or Bcl-3 (an additional transcriptional co-activator). Notably, p50 and p52 proteins are derived from larger precursor molecules, p105 and p100, respectively, unlike other NF-κB family members [11].

NF-κB family members are inhibited by IκB proteins, which are located in the cytoplasm. Currently, seven IκB family members, IκBα, IκBβ, Bcl-3, IκBε, IκBγ, and the precursor proteins p100 and p105 were identified [12,13]. NF-κB was found in various mammalian cells such as macrophages of the kidney, liver, lung as well as the central nervous, gastrointestinal, and cardiovascular systems. It leads to the production of various cytokines and chemokines, such as interleukin (IL)-1, IL-2, IL-6, IL-12, TNF-α, lymphotoxin (LT)-α, LT-β, and granulocyte-macrophage colony-stimulating factor (GM-CSF). It was demonstrated that upon stimulation with an appropriate cytokine, phosphorylation of IκBs by IκB kinase leads to nuclear translocation of NF-κB, promoting the transcription of various genes involved in inflammation, cell proliferation, host immunity, and apoptosis [11,14,15]. NF-κB therefore serves as a ‘rapid acting’ primary transcription factor, which can regulate diverse cellular responses such as the host’s early innate immune response to infection, and is also associated with chronic inflammatory conditions, viral infections, and multi-organ failure [16,17]. Moreover, the constitutive activation of NF-κB pathways was shown in inflammatory diseases such as multiple sclerosis and rheumatoid arthritis [10].

Multiple studies have shown that the NF-κB pathway consists of canonical and non-canonical pathways [14,16]. The canonical NF-κB is activated by various stimuli, leading to a rapid but transient transcriptional activity. It regulates the expression of many pro-inflammatory genes and serves as a crucial mediator for inflammatory responses [18]. In contrast, the non-canonical NF-κB pathway is activated through tumour necrosis factor (TNF) receptor family members [16]. Since the activation of this pathway requires protein synthesis, the kinetics of non-canonical NF-κB activation is slow but persistent, in accordance with its biological role in immune homeostasis and immune response. Both the canonical and non-canonical NF-κB pathways are tightly regulated, reflecting the important role of ubiquitination for their activation [19]. Recent studies suggest that dysregulated NF-κB activity contributes to inflammation-related diseases, and NF-κB has long been proposed as a potential therapeutic target [12,20,21].

The non-canonical pathway is mediated through TNF-α, which serves as a pro-inflammatory cytokine that activates NF-κB, MAPK, and PI3K/AKT pathways and plays a central role in inflammation, immune system development, and apoptosis. The inhibition of TNF-α was successful in treating many autoimmune disorders [22].

Macrophages and activated T cells are the primary producers of TNF-α in response to inflammation and infection [20,21].

Cellular responses to TNF-α are mediated through interaction with two receptors, TNF-R1 and TNF-R2, and result in the activation of pathways that favour both cell survival and apoptosis depending on the cell type and biological context [22].

In our recent paper, we described that the Janus kinase signal transducer and activator of the transcription (JAK-STAT) signalling pathway is activated in response to various cytokines and that this pathway is functional in MG-63 cells upon cultivation on both titanium and zirconia surfaces [23]. The purpose of this in vitro study was to investigate the activation of the NF-κB pathway upon stimulation with TNF-α in MG-63 cells cultured on zirconia surfaces in comparison to titanium surfaces.

## 2. Materials and Methods

### 2.1. Materials

DMEM and RPMI-1640 media for cell culture, fetal calf serum (FCS), trypsin-EDTA, and cell culture supplements were purchased from Bioconcept (Allschwil, Switzerland). Human recombinant tumour necrosis factor alpha (TNF-α) and human recombinant interferon gamma (IFN-γ) were from R&D Systems (Minneapolis, MN, USA). Primary rabbit monoclonal antibody recognizing human phosphorylated nuclear factor kappa B (NF-κB) protein was obtained from Cell Signaling (Danvers, MA, USA), while the antibody recognizing unphosphorylated NF-κB (p65) was from Santa Cruz (CA, USA). Mouse monoclonal antibodies against human vinculin and human actin for immunofluorescence experiments were obtained from Sigma/Merck (Buchs, Switzerland). Secondary antibodies conjugated with Alexa Fluor 488 (goat anti-mouse) and Alexa Fluor 555 (goat anti-rabbit) were from Merck (Buchs, Switzerland). For flow cytometry, the following antibodies were applied: fluorescein isothiocyanate (FITC)-conjugated mouse anti-human monoclonal antibodies against major histocompatibility complex (MHC) class I human leukocyte antigen-A,B,C (HLA-A,B,C), and FITC-conjugated mouse anti-human HLA-DR and HLA-DQ, as well as isotype-matched IgG from BD Biosciences (San Jose, CA, USA). All other chemicals utilized in this study were from Merck (Buchs, Switzerland) and of the highest purity grade. Cell culture experiments were performed in TPP plastic ware (Trasalingen, Switzerland). Zirconia and titanium discs were prepared as it was previously described in detail [24,25]. Briefly, machined titanium disc (T) with a polished surface obtained through a grinding process, which corresponds to the clinically standardized polished and smooth regions typically used for tissue-level dental implants, and polished zirconia discs (Z), which corresponds to the polished part of the Straumann^®^ PURE CERAMIC MONOTYPE implant. All tested control groups were manufactured and provided by the Straumann Group (Basel, Switzerland).

### 2.2. Cell Culture

Human osteosarcoma cell line MG-63 was from ATCC (CRL-1427) and cells were grown in Dulbecco’s minimal essential medium (DMEM) supplemented with 10% FCS and 1% Kanamycin Solution at 37 °C, 5% CO_2_ according to corresponding tissue culture collection protocol. Human Wilms tumour cell line WT-CLS1 was from Cell Lines Services (CLS GmBH, Eppelheim, Germany) and grown in RPMI-1640 medium with supplements. All cell lines were accompanied by identification test certificates and were grown according to corresponding tissue culture protocols.

### 2.3. Western Blot

Western blot (immunoblot) analysis of phospho-p65 and unphosphorylated p65 protein was performed as previously described [23]. In brief, cells were treated with TNF-α (10 ng/mL) for 20 min at 37 °C or left untreated (negative control), then lysed in a lysis buffer (50 mM Tris-HCl, 5 mM EDTA, 150 mM NaCl, 0.5% Nonidet-40, 1 mM PMSF, 10 µM sodium vanadate, and protein inhibitors aprotinin, leupeptin and pepstatin (1 µg/mL each), pH 8.0) on ice for 30 min. After centrifugation for 5 min at full speed, 40 µg total protein was mixed with 4x NuPAGE LDS loading buffer, 10x NuPAGE sample reducing agent containing DTT (dithiothreitol) from Invitrogen (Carlsbad, CA, USA), and resolved on NuPAGE Novex 4–12% Bis-Tris gels. The proteins were transferred to a nitrocellulose membrane using Novex Tris-Glycine Transfer buffer (Invitrogen, Carlsbad, CA, USA) according to the manufacturer’s instructions. Nonspecific binding sites were blocked with 5% milk in TBST (120 mM Tris-HCl, pH 7.4, 150 mM NaCl, and 0.05% Tween 20) for 1 h at room temperature. Target proteins were detected using specific antibodies for phospho-p65 or total p65 protein, see Materials Section 2.1. The membranes were washed three times and incubated with secondary antibodies conjugated with horseradish peroxidase. Immune complexes were visualized using the enhanced chemiluminescence system (Bio-Rad, Hercules, CA, USA). Data are representative of three independent experiments with nearly identical results. Whole cell extracts from WT-CLS1 cells were used as a control for immunoblots [26].

### 2.4. Immunofluorescence Staining

The MG-63 cells were cultured on Z or T discs for 72 h in a 24-well plate. In addition, the MG-63 cells were grown on 12 mm cover glasses. The cells were either left untreated (negative control) or incubated with TNF-α for 20 min at 37 °C. Afterwards, the cells were washed and fixed in ice-cold methanol/acetone (1:1) for 20 min, then incubated with 10% goat serum in PBS for 1 h at room temperature. Mouse anti-human vimentin or mouse anti-human β-actin antibodies were used as primary antibodies. Goat anti-mouse Alexa Fluor 488 or goat anti-rabbit Alexa Fluor 555 (Sigma/Merck, Buchs, Switzerland) were used as secondary antibodies. The nuclei were stained with DAPI. The images were captured and analyzed on an Olympus BX-51 microscope with 40x objective using proprietary software, as described before [27]. Three independent experiments were performed.

### 2.5. MHC Class I and Class II Modulation Assay

The surface expression of MHC class I and class II was monitored by flow cytometry, using a Peridinin-Chlorophyll Protein Complex (PerCP)-labelled mouse anti-human monoclonal antibody for HLA-A, B, C heavy chain and fluorescein isothiocyanate (FITC)-conjugated mouse anti-human antibodies against MHC class II (HLA-DR, HLA-DQ) or control, isotype-matched reagent in cells cultured for 48 h in the presence or absence of TNF-α and IFN-γ, as previously described [23]. The mean fluorescence intensity of stained cells was measured and analyzed using a FACS Calibur analyser and the CellQuest Pro Software (Version 5.2.1).

### 2.6. MTT Cell Viability Assay

Thirty thousand MG-63 cells were cultured on T and Z discs in 24-well cell culture plates, or in wells without any discs. The cells were incubated for 72 h, followed by 48 h incubation with TNF-α. MTT (thiazolyl blue tetrazolium bromide) was added at a concentration of 0.1 mg/mL, and the cells were incubated for 4 more hours. The reaction was stopped by adding 125 µL of DMSO. The supernatants were harvested, and optical density was measured at 590 nm, as previously described [28]. Three independent experiments were performed in triplicates.

### 2.7. Statistical Analysis

Each experiment was performed at least three times in multiple replicates and statistical analysis was conducted using a two-tailed Student’s *t*-test. The data are reported as means ± standard deviation. Statistical probabilities (*p*) were expressed as * when *p* < 0.05, ** when *p* < 0.01, and *** when *p* < 0.001. Data were considered significant when *p* < 0.05.

## 3. Results

To investigate the effects of TNF-α on MG-63 cells cultured on titanium (T) and zirconia (Z) surfaces, immunoblot, fluorescent immunostaining, MTT cell viability, and flow cytometry experiments were performed.

### 3.1. Western Blot Analysis of Phosphorylated NF-κB Protein p65

**Figure 1:**
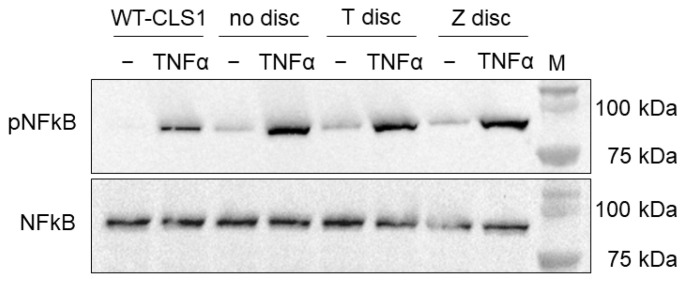
WT-CLS1 and MG-63 cells were treated with human recombinant TNF-α. Membranes were probed with anti-phospho-p65 recognizing only phosphorylated p65. In addition, membranes were also probed with corresponding NF-κB/p65 antibody, which recognizes total p65 independent of phosphorylation. Lysates of WT-CLS1 cells treated with human recombinant TNF-α were used as positive control.

The MG-63 cells were incubated on T or Z discs and on cell culture wells without any discs as a control. The cells were treated with a human recombinant TNF-α as outlined in Section 2 or left untreated as a negative control. As shown in Figure 1, a strong activation of phopsho-p65 protein was observed in all TNF-α treated samples. Protein lysates of WT-CLS1 were used as a positive control of phospho-p65 expression [26]. As shown in Figure 1 (upper panel), all examined samples express constitutively phospho-p65, but its expression substantially increased after TNF-α stimulation. The lysates of the WT-CLS1 and MG-63 cells were also probed with antibodies, which recognize p65 independent of phosphorylation (Figure 1, lower panel). All samples independent of TNF-α treatment express high amounts of p65. We also did not observe any differences in p65 expression when the cells were cultivated on T or Z discs or without any discs.

### 3.2. Immunofluorescence Analysis of Vinculin and Actin in MG-63 Cells

**Figure 2:**
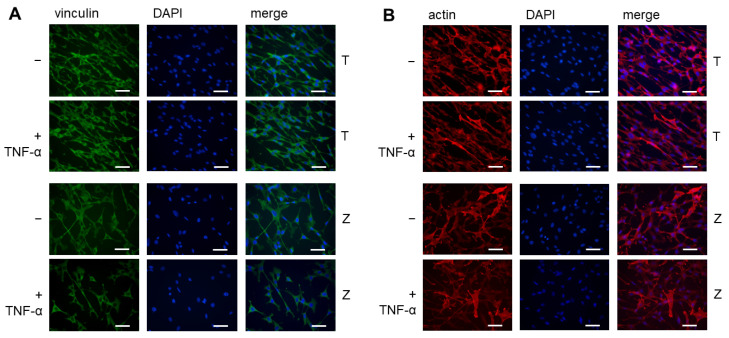
Immunofluorescence analysis of vinculin (**A**) and actin (**B**) expression in MG-63 cells treated or untreated with TNF-α. Cytoplasmic vinculin staining is shown in green, actin staining in red and nuclear DAPI staining in blue. Cells were cultured for 72 h on T or Z discs. In all images, magnification is 40x, scale bar—50 μm. Images are from one experiment representative of three, which gave very similar results.

The expression of actin and vinculin in MG-63 cells after incubation on T and Z discs was also analyzed by immunofluorescence microscopy. As shown in Figure 2A, the cells were incubated on T discs or Z discs, treated with TNF-α or left untreated as a negative control, and stained with anti-vinculin antibody (green). In Figure 2B, the MG-63 cells were incubated on T or Z discs, treated with TNF-α, and stained with anti-actin antibody (red).

In all examined stimulated and unstimulated cells, a cytoplasmic vinculin and actin staining was visible. We did not observe any differences in the expression levels of both vinculin and actin in TNF-α-stimulated cells compared to unstimulated cells. There are also no substantial differences between cells cultured on T or Z discs.

### 3.3. Flow Cytometry Analysis of MHC Class I and Class II Modulation

**Figure 3:**
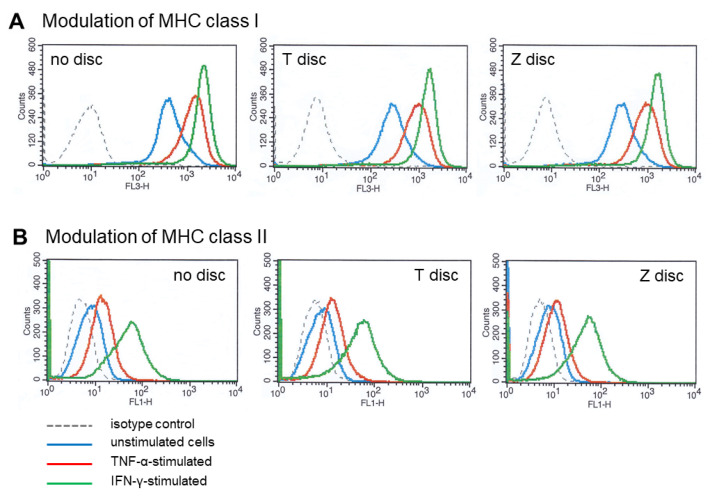
(**A**) MHC class I modulation. Flow cytometry analysis of MG-63 cells cultivated for 72 h on T or Z discs and then stimulated with TNF-α or IFN-γ for 48 h. MG-63 cells express high amounts of MHC class I (blue histograms). Stimulation with TNF-α or IFN-γ led to overexpression of MHC class I. Grey dotted histograms represent isotype-matched negative controls. (**B**) MHC class II modulation. Flow cytometry analysis of MG-63 cells cultivated for 72 h on T or Z discs and then stimulated with TNF-α or IFN-γ for 48 h. Expression of MHC class II was analyzed. After stimulation with IFN-γ, MG-63 cells overexpress MHC class II proteins in all examined cells. Stimulation with TNF-α slightly increased MHC class II expression compared to strong shift observed after IFN-γ stimulation. Grey dotted histograms represent isotype-matched negative controls.

The response to human recombinant TNF-α on MG-63 cells was also analyzed by flow cytometry. The cells were cultured on T or Z surfaces for 72 h, then the cells were treated for 48 h with TNF-α or IFN-γ, or left untreated as a negative control. The expression of MHC class I and MHC class II proteins was analyzed by flow cytometry. As shown in Figure 3A, untreated MG-63 cells express high levels of MHC class I (blue histograms). When the cells were treated with 10 ng/mL of TNF-α, the upregulation of MHC class I expression on both T and Z discs as well as on wells without any discs was observed (red histograms). As a positive control for the upregulation of MHC class I, the MG-63 cells were also treated with IFN-γ, which induced a strong upregulation of MHC class I proteins (green histograms).

As shown in Figure 3B, only stimulation with IFN-γ on MG-63 cells caused an upregulation of MHC class II expression in all samples (green histograms) whereas cells treated with TNF-α have shown a minor upregulation of MHC class II expression (red histograms). Unstimulated cells do not express MHC class II (blue histograms). The grey histograms represent the isotype-matched controls (Figure 3A,B).

### 3.4. Effect of TNF-α in MTT Assay

**Figure 4:**
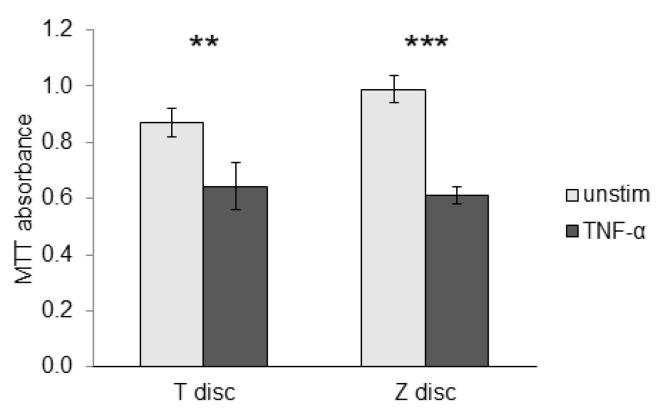
MG-63 cells were incubated with TNF-α as outlined in Materials and Methods. TNF-α significantly suppressed metabolic activity of cells cultured on T and Z surfaces. Values represent mean ± SD. Three independent experiments were performed in triplicates. Statistical probabilities (*p*) were expressed as ** when *p* < 0.01 and *** when *p* < 0.001.

To evaluate the role of TNF-α on the proliferative capacity of MG-63 cells, we further performed an MTT assay. As shown in Figure 4, TNF-α significantly suppressed the metabolic activity of MG-63 cells when they were incubated on T and Z surfaces (black columns) compared to unstimulated cells (grey columns).

### 3.5. Western Blot Analysis of Alkaline Phosphatase

**Figure 5:**
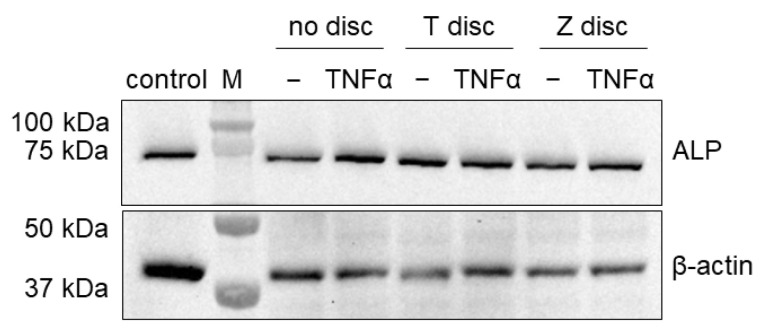
MG-63 cells were treated with human TNF-α as outlined in Materials and Methods. Membranes were probed with anti-ALP antibody (**upper part**). In addition, membranes were probed with β-actin antibody (**lower part**).

We also investigated if TNF-α alters the activity of alkaline phosphatase (ALP) of MG-63 cells grown on T and Z surfaces. As shown in Figure 5, no differences were observed on the expression levels of ALP when MG-63 cells were cultivated on T or Z discs or without any discs. Incubation with TNF-α did not have any influence on ALP expression. In addition, we also probed the membranes with antibodies against β-actin. As shown in Figure 5 (lower panel), all cells expressed β-actin and we did not observe any differences in the expression levels between the samples.

## 4. Discussion

In dentistry, zirconia implants are a promising alternative to the well-established titanium implants. Zirconia implants may offer several biological benefits including enhanced soft tissue adhesion, inhibition of bacterial adhesion, and improved osseointegration [8,29]. It was reported that the success rate after five years was notably high [5,8,9], though it is important to note that this period may be too short for a comprehensive evaluation. The comparison between zirconia and titanium and their respective influence on the immune response however requires further investigation. As more clinical data become available, the choice of implant material will still remain crucial. The tissues and cells surrounding the implant must be able to respond effectively to bacteria, cytokines, and other factors to promote successful implantation. In this regard, the use of zirconia implants might be advantageous compared to titanium since it permits a greater inhibition of bacterial adhesion. However, both zirconia and titanium are known to offer similar capabilities with regard to soft tissue adhesion [30].

Cytokines play very important roles in many processes including inflammation, cardiovascular diseases, cancer progression, autoimmune diseases, and neurodegenerative disorders. The activation of the JAK-STAT signalling pathway in response to various cytokines in MG-63 cells cultivated on the surface of either titanium or zirconia was previously characterized in detail [23]. In the current study, however, the focus was on another very important signalling pathway. We investigated whether the NF-κB signalling pathway is activated in MG-63 cells cultured on both titanium and zirconia surfaces. Zirconia discs were compared to classical titanium discs, and the activation of the NF-κB pathway was studied using several methods including immunoblot, fluorescent immunostaining, MTT assays, and flow cytometry.

Our data demonstrated that the NF-κB signalling pathway was unimpaired in MG-63 cells grown on either titanium or zirconia surfaces. Western blot data revealed that human recombinant TNF-α caused a strong activation of phospho-p65. This activation was observed in all samples, and the type of surface, whether titanium, zirconia, or commercial cell culture wells, had no impact on this effect.

The immunofluorescence data showed that the expression patterns of actin and vinculin were very similar when MG-63 cells were cultured on either titanium or zirconia surfaces. We did not observe any significant differences between them.

In the next set of experiments, we investigated the effects of human recombinant TNF-α on the expression of MHC class I and class II proteins, and compared the expression levels to those observed after stimulation with IFN-γ. We previously demonstrated that human recombinant IFN-γ strongly activates the STAT1 protein via the JAK-STAT pathway in MG-63 cells cultivated on titanium and zirconia surfaces [23]. IFN-γ causes a strong upregulation of MHC class I and class II proteins in MG-63 cells. Incubation with TNF-α led to a significant upregulation of MHC class I proteins in MG-63 cells grown on the titanium and zirconia discs, but this response was less strong compared to the IFN-γ stimulation. Our data from MG-63 cells cultivated on titanium and zirconia discs, as well as in commercial cell culture wells without any discs, showed very similar results in response to TNF-α and IFN-γ. A very weak upregulation of MHC class II proteins after stimulation with TNF-α was detected in all samples compared to a stronger overexpression of MHC class II proteins after stimulation with IFN-γ.

Our MTT data did not reveal significant differences in the metabolic activity between the titanium and zirconia discs when we compare unstimulated cells with each other, as well as when the activity of TNF-α-stimulated cells are compared with each other. In contrast, reduced metabolic activity was observed in MG-63 cells cultivated on titanium and zirconia surfaces after stimulation with TNF-α when these are compared to unstimulated cells, respectively. The detailed mechanisms underlying the interactions between MG-63 cells and titanium or zirconia surfaces in vitro remain unknown. These observed effects might be caused by the cytotoxic effects of the titanium or zirconia surfaces on cells, which was reported previously [31,32].

We also investigated the expression of alkaline phosphatase (ALP) in MG-63 cells cultured on titanium and zirconia surfaces. ALP promotes the hydrolysis of phosphate-containing substrates, increases the uptake of calcium, and is considered a periodontal disease marker [33]. Abnormalities in ALP expression were detected in dental pulps, certain types of cancers, and in inflammatory diseases [34,35]. Our Western blot data showed that ALP expression levels are similar when titanium or zirconia discs are used, and the stimulation of cells with TNF-α did not have any visible effect on this expression.

All experiments were performed in vitro using MG-63 cells. As with any study involving a cell line, this model has limited generalizability due to its restricted exploration of heterogeneity.

In summary, our data have demonstrated the ability of both titanium and zirconia to support cell growth, attachment, and proliferation of MG-63 cells. The activation of the NF-κB signalling pathway in response to cytokines was very similar on both surfaces.

Ten years ago, the Straumann Group (Basel, Switzerland) has developed and patented a titanium–zirconium alloy known for its excellent biocompatibility and osseointegration properties. This product was branded as Roxolid^®^ [36]. Roxolid^®^ implants showed very promising results regarding its stability compared to widely used titanium implants and could be a better alternative [37,38].

## 5. Conclusions

In this current study, NF-κB pathway activation in MG-63 cells was demonstrated using Western blot, MTT assay, flow cytometry, and immunofluorescence techniques. Upon stimulation with TNF-α, the NF-κB pathway was activated in the MG-63 cells cultured on both titanium and zirconia surfaces. The NF-κB signalling pathways could be activated in osteoblast-like MG-63 cells on both zirconia and titanium surfaces in response to cytokines.

## Data Availability

The original contributions presented in the study are included in the article, further inquiries can be directed to the corresponding author.
